# Immunosuppressive agents for frequently relapsing/steroid-dependent nephrotic syndrome in children: a systematic review and network meta-analysis

**DOI:** 10.3389/fimmu.2024.1310032

**Published:** 2024-02-23

**Authors:** Yu Zhu, Junyi Chen, Yao Zhang, Xiaoai Wang, Jingjing Wang

**Affiliations:** ^1^ Department of Traditional Chinese Medicine, Children’s Hospital, Zhejiang University School of Medicine, National Clinical Research Center for Child Health, Zhejiang, Hangzhou, China; ^2^ Department of Nephrology, Children’s Hospital, Zhejiang University School of Medicine, National Clinical Research Center for Child Health, Zhejiang, Hangzhou, China

**Keywords:** immunosuppressive agent, rituximab, frequently relapsing/steroid dependent nephrotic syndrome, children, network meta-analysis

## Abstract

**Aim:**

This study aimed to systematically compare the efficacy of various immunosuppressive agents in treating pediatric frequently relapsing or steroid-dependent nephrotic syndrome (FRSDNS).

**Methods:**

We conducted systematic searches of PubMed, Embase, the Cochrane Library, and the Web of Science up to May 23, 2023. Outcome measures included relapses within 1 year, mean cumulative exposure to corticosteroids, patients with treatment failure at 1 year, relapse-free survival during 1 year, and adverse events. The quality of the included studies was evaluated using the modified Jadad scale, the Methodological Index for Non-Randomized Studies (MINORS), and the modified Newcastle-Ottawa Scale (NOS).

**Results:**

Rituximab was found to be the most likely (92.44%) to be associated with the fewest relapses within 1 year and was also most likely (99.99%) to result in the lowest mean cumulative exposure to corticosteroids. Rituximab had the highest likelihood (45.98%) of being associated with the smallest number of patients experiencing treatment failure at 1 year. CsA was most likely (57.93%) to achieve the highest relapse-free survival during 1 year, followed by tacrolimus (26.47%) and rituximab (30.48%). Rituximab showed no association with serious side effects and had comparable adverse effects to ofatumumab and tacrolimus.

**Conclusion:**

Rituximab may be the most favorable immunosuppressive agent for treating pediatric FRSDNS. Nephrologists should consider this drug, along with their clinical experience, patient characteristics, and cost considerations, when choosing a treatment approach.

## Introduction

Nephrotic syndrome plays a significant role in the progression to end-stage kidney disease (1), characterized by several key features, including edema, proteinuria, low blood albumin levels, and elevated lipid levels in the blood (2). As the most common glomerular disease affecting children, nephrotic syndrome impacts between two and five children per one hundred thousand each year (3). Corticosteroids serve as the primary treatment for idiopathic nephrotic syndrome (4), however, it is estimated that around 50% of children diagnosed with steroid-sensitive nephrotic syndrome (SSNS) will experience frequent relapsing or steroid-dependent nephrotic syndrome (FRSDNS) (5). Immunosuppressive drugs have proven effective in treating children with FRSDNS (6–8).

Immunosuppressive medications used to treat pediatric FRSDNS include rituximab, cyclophosphamide, tacrolimus, and mycophenolate mofetil (MMF). A recent study showed that children with FRSDNS who were treated with rituximab experienced improved one-year relapse-free survival, fewer relapses, and reduced cumulative corticosteroid exposure compared to those treated with cyclophosphamide and tacrolimus (9). In pediatric steroid-dependent nephrotic syndrome (SDNS), treatment with MMF/dexamethasone (DEX) has been shown to be more effective than cyclosporine A (CsA) in reducing relapses and cumulative corticosteroid dosage (10). According to another study, children with SDNS treated with ofatumumab and rituximab experienced similar rates of relapses and adverse events (11). A 2019 network meta-analysis aimed at identifying the best immunosuppressive drug for children with FRSDNS suggested that cyclophosphamide might initially be the most suitable option (6). Nonetheless, the study was limited by its inclusion of placebo and no treatment as controls, insufficient direct comparisons between immunosuppressive drugs, and neglect of recent literature updates.

To address these research gaps, our network meta-analysis aimed to systematically assess and rank the effectiveness of different immunosuppressive drugs in treating pediatric FRSDNS, drawing on the latest direct comparison evidence among these agents.

## Methods

### Search strategy

Four electronic databases (PubMed, Embase, Cochrane Library, and Web of Science) were systematically searched by two independent investigators (JY Chen, Y Zhang) from database inception until May 23, 2023. Discrepancies were addressed via discussion. The following English search terms were applied: “Immunosuppressive Agents” OR “Agents, Immunosuppressive” OR “Immunosuppressants” OR “Immunosuppressive Agent” OR “Agent, Immunosuppressive” OR “Immunosuppressant” OR “Immunosuppressive drugs” OR “Cyclophosphamide” OR “Cyclosporine” OR “Cyclosporine A” OR “Tacrolimus” OR “Mycophenolate mofetil” OR “MMF” OR “Rituximab” OR “Cyclosporin” OR “Azathioprine” OR “Mizoribine” OR “Vincristine” OR “Calcineurin inhibitors” OR “Mycophenolic sodium” OR “MPS” AND “Nephrotic Syndrome” OR “Nephrotic Syndromes” OR “Syndrome, Nephrotic” OR “Steroid-Dependent Nephrotic Syndrome” OR “Nephrotic Syndrome, Steroid-Dependent” OR “Steroid Dependent Nephrotic Syndrome” OR “Steroid-Dependent Nephrotic Syndromes” OR “Childhood Idiopathic Nephrotic Syndrome” OR “Pediatric Idiopathic Nephrotic Syndrome” OR “Multi-Drug Resistant Nephrotic Syndrome” OR “Multi Drug Resistant Nephrotic Syndrome” OR “Steroid-Sensitive Nephrotic Syndrome” OR “Nephrotic Syndrome, Steroid-Sensitive” OR “Steroid Sensitive Nephrotic Syndrome” OR “Steroid-Sensitive Nephrotic Syndromes” OR “Syndrome, Steroid-Sensitive Nephrotic” OR “Steroid-Resistant Nephrotic Syndrome” OR “Nephrotic Syndrome, Steroid-Resistant” OR “Steroid Resistant Nephrotic Syndrome” OR “Steroid-Resistant Nephrotic Syndromes” OR “Frequently Relapsing Nephrotic Syndrome” OR “Frequent relapses or steroid dependence nephrotic syndrome” OR “FRSDNS”. Initial screening of the retrieved studies was performed by reviewing titles and abstracts, followed by full-text evaluations. This systematic review and network meta-analysis adhered to the guidelines outlined in the Preferred Reporting Items for Systematic Reviews and Meta-Analyses (PRISMA) extension statement for network meta-analyses.

### Eligibility criteria

The inclusion criteria were structured according to the Population, Intervention, Comparator, Outcome, Study design (PICOS) framework, starting with: (1) studies focusing on children diagnosed with FRSDNS (Population; (2) studies that investigated the treatment of pediatric FRSDNS with various immunosuppressive drugs, including rituximab, cyclophosphamide, tacrolimus, MMF, CsA, and ofatumumab (Intervention and Comparator); (3) studies on at least one of the following outcomes: relapses within 1 year, mean cumulative exposure to corticosteroids (mg/kg/year), patients with treatment failure at 1 year, relapse-free survival during 1 year, and adverse events (Outcome); (4) clinical controlled trials and cohort studies (Study design); (5) studies published in English language.

Exclusion criteria: (1) studies involving animal experiments; (2) studies using placebo or prednisone as the control group; (3) studies with fewer than 10 participants; (4) studies focused on comparing different doses of the same drug; (5) studies examining different methods of medication administration; (6) studies unrelated to the topic; (7) case reports, editorial materials, meeting abstracts, protocols, guidelines, expert consensus, reviews, and meta-analyses.

### Data extraction and quality assessment

Two investigators (JY Chen, XA Wang) extracted the following data independently from eligible studies: first author, year of publication, country, study design, population, intervention, immunosuppressive agents, sample size, male/female, age (years), proteinuria, urine protein/creatine ratio, albumin (g/dL), follow-up time (months), and outcome. Disagreements were settled by another investigator (Y Zhang).

### Quality assessment

The quality of randomized controlled trials was assessed using the modified Jadad scale, which evaluates four key aspects: the generation of random sequences, concealment of randomization, the methodology of blinding, and the withdrawals and dropouts (12). The modified Jadad scale has a total possible score of 7 points. Studies scoring between 1 and 3 points are considered to be of low quality, while those achieving a score of 4 to 7 points are classified as high quality. The quality of non-randomized controlled trials was evaluated using the Methodological Index for Non-Randomized Studies (MINORS), which has 12 items for comparative studies, with each item scoring 0-2 points (0: not reported; 1: reported but inadequate; 2: reported and adequate) (13). The quality of cohort studies was assessed using the modified Newcastle-Ottawa Scale (NOS), which evaluates three primary aspects: selection of the population, comparability between groups, and measurement of outcomes. The total score on this scale can reach up to 9 points. Studies scoring between 0 and 3 points were considered low quality, those with 4 to 6 points were deemed medium quality, and studies achieving 7 to 9 points were categorized as high quality (14).

### Statistical analysis

Using Gemtc 1.0.1 package from R 4.1.3 (R Foundation for Statistical Computing, Vienna, Austria), a Bayesian framework and Monte Carlo Markov Chain (MCMC) model were constructed for network meta-analysis. The model has 4 chains, 20000 initial iterations, and 50000 updated iterations with a step size of 1. When there was a network relationship, consistency and inconsistency were detected in the direct and indirect comparisons. When the difference in the Deviation Information Criterion (DIC) between the results of consistency and inconsistency detection was less than 5, it indicated that the strength of the direct and indirect evidence was consistent. The heterogeneity test was conducted for the effect size of each outcome. If the heterogeneity statistic I^2^ ≥ 50%, a random-effects model was used for analysis. Otherwise, a fixed-effects model was utilized. Network plots, forest plots, league tables, and rank probabilities were depicted for each outcome. For network plots, nodes represented different immunosuppressive agents, while the lines linking these nodes represented the direct comparisons between the agents. The size of nodes and the thickness of lines showed the sample size and the number of studies included for comparison, respectively. Weighted mean differences (WMDs) along with 95% credibility intervals (CrIs) were used to present measurement data. For enumeration data, odds ratios (ORs) and their corresponding 95% credibility intervals (95%CrIs) were calculated.

## Results

### Study selection

**Figure 1:**
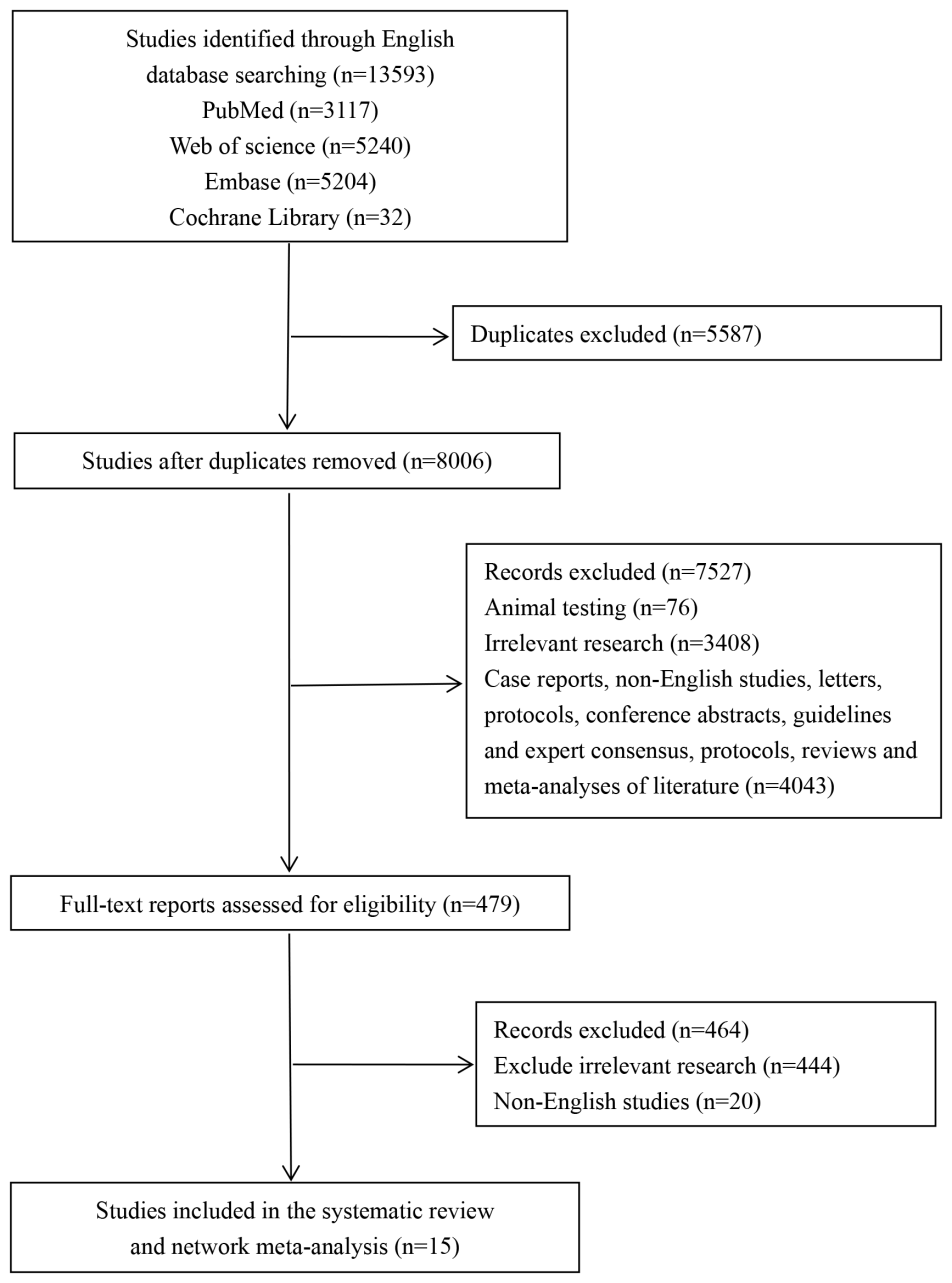
Flow chart of study selection.

Through systematic searches across four databases, a total of 13,593 studies were retrieved, including 3,117 from PubMed, 5,240 from Embase, 5,204 from the Cochrane Library, and 32 from Web of Science. After excluding 5,587 duplicates and 7,527 studies based on their titles and abstracts, 479 studies remained for full-text evaluation. Eventually, 15 studies (9–11, 15–26) were included in this systematic review and network meta-analysis. The study selection process is depicted in **Figure 1**.

### Characteristics of the included studies

The 15 eligible studies (9–11, 15–26) included 1053 pediatric patients with FRSDNS. The year of publication covered 2008 to 2022, and 9 countries were involved. A total of 6 immunosuppressive agents were assessed in these studies: rituximab, cyclophosphamide, tacrolimus, MMF, CsA, and ofatumumab. There were 5 randomized controlled trials, 1 non-randomized controlled trial, and 9 cohort studies. The quality assessment results indicated that 12 studies were of high quality, 2 were of low quality, and one study achieved a MINORS score of 18 points. The characteristics of the included studies are detailed in **Table 1**.

**Table 1:** Characteristics of the included studies.

Author	Year	Country	Study design	Population	Intervention	Immunosuppressive agent	Sample size	M/F	Age (years)	Proteinuria	Urine protein/creatine ratio	Albumin (g/dL)	Follow-up time (months)	Outcomes	QA
Wang	2022	China	RCT	Children with FRSDNS	Cyclophosphamide	Cyclophosphamide	17	NA	<18	NA	NA	NA	12	Relapses, infection, the cumulative prednisolone dosage	3
					Tacrolimus	Tacrolimus	17	NA		NA	NA	NA	12		
					Rituximab	Rituximab	17	NA		NA	NA	NA	12		
Tunçay	2021	Turkey	Cohort study	Patients diagnosed with SDNS	MMF (25–36 mg/kg/day, maximum 2 g/day) given in twoequal daily doses	MMF	29	17/12	9.93 ± 4.23	151.60 ± 185.87(mg/m^2^/h)	NA	NA	36	Annual relapse rates, the number of annual relapses and cumulative steroid dosage	8
					The CsA group, received CsA at a dose of 3–5 mg/kg/day, divided into two daily doses	CsA	25	13/12	5.16 ± 1.52	187.60 ± 218.72(mg/m^2^/h)	NA	NA	36		
Ravani	2021	Italy	RCT	Children with SDNS	Ofatumumab was infused intravenously, at a dose of 1500 mg/1.73 m2, diluted in 1000 ml of normal saline, at a speed of 12 ml/hour in the first 30’.	Ofatumumab	70	44/26	10 (6–16)	80 (60–130) (mg/day)	NA	4 ± 1	24	Relapse at 12 months	5
					Rituximab was administered at a dose of 375 mg/m^2^ diluted in the required volume of saline to achieve the desired concentrations	Rituximab	70	52/18	11 (5–15)	75 (50–120) (mg/day)	NA	4 ± 1	24		
Kari	2020	Kingdom of Saudi Arabia	Non-RCT	Pediatric patients diagnosed with idiopathic FRSDNS	Rituximab	Rituximab	19	14/5	7.18 ± 3.36	NA	NA	NA	12	Relapse-free survival during 1-year, steroid withdrawal within 3 months, reducing the prescribed dose of oral alternate day steroid	18
					Cyclophosphamide	Cyclophosphamide	27	13/14	5.68 ± 2.75	NA	NA	NA	12		
Solomon	2019	India	RCT	Patients aged 3–16 years with SDNS	Tacrolimus: 0.2 mg/kg/day (target trough levels of 5–7 ng/mL).	Tacrolimus	60	NA	13-16(range)	NA	NA	NA	12	Patients with sustained remission over 12 months, the relapse rate	3
					Rituximab: T wo to four infusions, until B cell depletion (one per week, dose 375 mg/m^2^, maximum 500 mg).	Rituximab	60	NA	13-16(range)	NA	NA	NA	12		
Mizutani	2019	Japan	Cohort study	Children with SDNS	Patients had undergone single daily high-dose MZR therapy from 2008 as maintenance therapy after cyclophosphamide therapy.	MZR + cyclophosphamide	36	28/8	5.8(mean)	NA	NA	NA	66.24 ± 20.16	Sustained remission over 2 years	7
					Patients had undergone cyclophosphamide monotherapy.	Cyclophosphamide	18	15/3	5.8(mean)	NA	NA	NA	126.84 ± 44.46		
Basu	2018	India	RCT	Patients with corticosteroid-dependent nephrotic syndrome	In the tacrolimus arm, children received tacrolimus, 0.2 mg/kg/d, targeting trough levels of 5 to 7 ng/mL along with taper-ing doses of alternate-day prednisolone.	Tacrolimus	60	32/28	7.2 ± 2.8	NA	NA	4.34 ± 0.81	12	Relapse-free survival rate, patients with treatment failure, serum albumin, cumulative prednisolone dose	5
					In the rituximab arm, children were scheduled to receive 2 to 4 rituximab infusions at weekly intervals (375 mg/m^2^, maximum dose, 500 mg) depending on the circulating B-cell count along with alternate-day prednisolone for 4 weeks.	Rituximab	60	32/28	7.1 ± 2.8	NA	NA	4.18 ± 0.73	12		
Basu	2017	India	Cohort study	Children who were treated for idiopathic FRSDNS	MMF 1200 mg/m^2^ every day in two divided doses.	MMF	130	84/46	7.1 ± 2.4	NA	NA	2.7 ± 1.6	30	Sustained remission, treatment failure, relapses per patient-year, cumulative prednisolone dose, serum albumin	9
					Tacrolimus 0.1–0.2 mg/kg (trough levels 5–7 ng/ml) every day in two divided doses along	Tacrolimus	81	54/27	6.6 ± 2.3	NA	NA	2.5 ± 1.4	30		
Alsaran	2017	Saudi Arabia	Cohort study	Children with idiopathic FRSDNS	Cyclophosphamide	Cyclophosphamide	12	NA	3.75 ± 1.1	NA	NA	1.8 ± 0.5	36	Relapse rate, cumulative dose of steroids, treatment failure	8
					MMF	MMF	15	NA		NA	NA		36		
					Cyclosporine	Cyclosporine	13	NA		NA	NA		36		
Wang	2016	China	Cohort study	Patients with primary SSNS	Administered MMF for 12 months, 20–30 mg/kg per day (two doses per day, maximum dose 1 g)	MMF	34	26/8	5.35 ± 2.68	NA	3.80 ± 3.97	2.494 ± 0.894	12	Relapse rate	9
					Administered TAC for 12 months, 0.05–0.15 mg/kg per day. The dose was adjusted to obtain trough levels of 5–10 μg/L.	Tacrolimus	38	25/13	6.01 ± 2.69	NA	3.81 ± 3.92	2.335 ± 1.166	12		
Wang	2012	China	Cohort study	Children with idiopathic FRSDNS	Initiated CsA treatment at 3–4 mg/kg/day, divided into two doses over 12-h intervals.The dose was adjusted according to each patient’s trough blood level, with a target of 100–150 ng/mL.	CsA	16	NA	7.7 ± 5.0	NA	NA	2.33 ± 2.01	24	Relapse rate, remission	9
					Initially administered TAC at 50–150 μg/kg/day, divided into two doses over 12-h intervals, and subsequently adjusted the dose according to each patient’s trough blood level, with a target of 5–12 ng/mL.	Tacrolimus	24	NA	8.6 ± 5.8	NA	NA	1.89 ± 1.58	24		
Sinha	2012	India	Cohort study	Children with SDNS	Rituximab was administered once weekly at a dose of 375 mg/m^2^ for two or three doses, as described previously with the aim of achieving CD19 depletion <1% of total lymphocyte count.	Rituximab	10	8/2	12.2 ± 2.3	NA	NA	3.8 ± 0.5	>12	Relapse rate, Remission, cumulative steroid dose	8
					Oral tacrolimus at a dose of 0.1–0.2 mg/kg/day in two divided doses for 12 months, targeting trough levels between 4 and 7 ng/ml.	Tacrolimus	13	10/3	12.3 ± 3.0	NA	NA	3.1 ± 0.9	>12		
Sümegi	2008	Hungary	Cohort study	Children with SDNS	CP was introduced at 2–2.5 mg/kg per day orally for 8–12 weeks.Mean duration of CP treatment was 2.5 ± 0.5 (2–3) months.	Cyclophosphamide	15	12/3	7.7 ± 3.8	4.1 ± 3.3 (g/24h)	NA	NA	60	Relapse-free period, relapse rate/year 5 years after therapy	8
					CSA was introduced at 3–5 mg/kg per day. Mean CSA treatment duration was 28 ± 15 (7–60) months.	CsA	8	7/1	9.5 ± 4.7	4.4 ± 2.6 (g/24h)	NA	NA	60		
Kranz	2008	Germany	Cohort study	Patients with SDNS	CsA treatment was started with a dosage of 100–150 mg/m^2^ per body surface area (BSA) in two divideddoses. CsA dose was adjusted to a target blood level of 80–120 ng/ml.	CsA	20	NA	8.4 ± 3.0	NA	NA	NA	5.4 ± 2.2	Glomerular filtration rate	7
					According to APN recommendations, these patients were treated with cyclophosphamide with 2 mg/kg body weight for 12 weeks	Cyclophosphamide	15	NA	6.0 ± 3.2	NA	NA	NA	4.9 ± 3.4		
Dorresteijn	2008	Netherlands	RCT	Children with FRNS	Treated for 12 months with MMF, 1200 mg/m^2^ per day b.i.d. (two doses per day, maximum dose 1 g twice daily)	MMF	12	10/2	10.4 ± 3.4	NA	NA	NA	12	relapse-free period	4
					CsA 4–5 mg/kg per day b.i.d. The dose was adjusted aiming at trough levels of 50–150 µg/l.	CsA	12	11/1	9.7 ± 4.2	NA	NA	NA	12		

M/F, male/female; QA, quality assessment; RCT, randomized controlled trial; FRSDNS, frequently relapsing or steroid-dependent nephrotic syndrome; SSNS, steroid-sensitive nephrotic syndrome; FRNS, frequently relapsing nephrotic syndrome; SDNS, steroid-dependent nephrotic syndrome; CsA; cyclosporine A; MMF, mycophenolate mofetil; MZR, mizoribine; APN, Arbeitsgemeinschaft Pädiatrische Nephrolgie; * standard deviation scores; NA, not applicable.

### Network analysis

#### Relapses within 1 year

**Figure 2:**
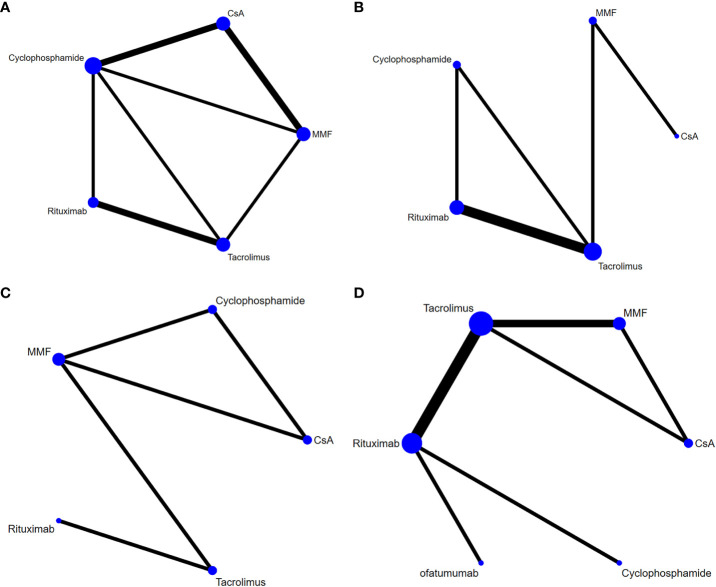
Network plots of different immunosuppressive agents for various outcomes in pediatric FRSDNS. **(A)**, relapses within 1 year; **(B)**, mean cumulative exposure to corticosteroids; **(C)**, patients with treatment failure at 1 year; **(D)**, relapse-free survival during 1 year. FRSDNS, frequently relapsing or steroid-dependent nephrotic syndrome; CsA; cyclosporine A; MMF, mycophenolate mofetil.

**Figure 3:**
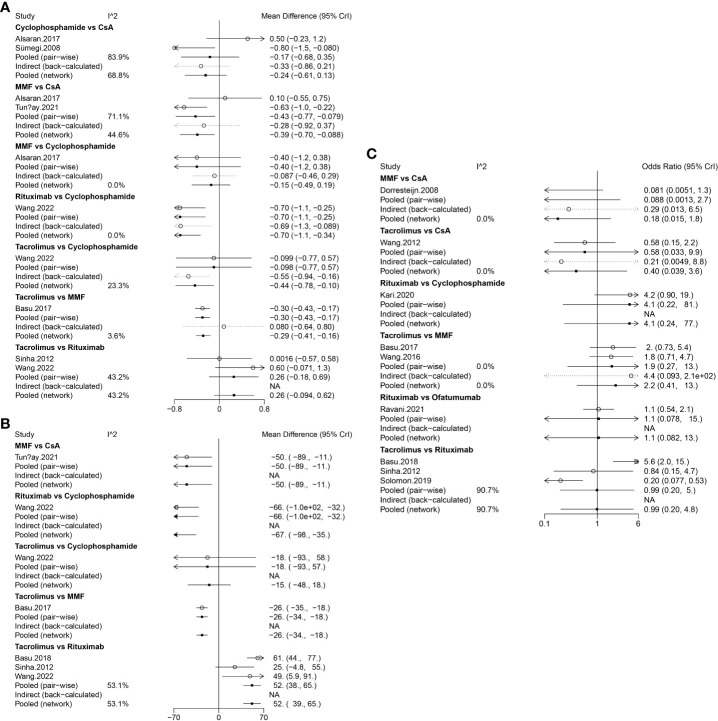
Forest plots of different immunosuppressive agents for various outcomes in pediatric FRSDNS. **(A)**, relapses within 1 year; **(B)**, mean cumulative exposure to corticosteroids; **(C)**, relapse-free survival during 1 year. FRSDNS, frequently relapsing or steroid-dependent nephrotic syndrome; CsA; cyclosporine A; MMF, mycophenolate mofetil; CrI, credibility intervals; NA, not applicable.

Data on relapses within 1 year were assessed in 6 studies (9, 10, 17, 18, 21, 22), involving 402 patients and 5 immunosuppressive agents (cyclophosphamide, rituximab, tacrolimus, MMF, and CsA). Cyclophosphamide was a more frequently used agent, and a relatively greater number of studies directly compared CsA and cyclophosphamide (**Figure 2A**). The forest plot revealed that children who received rituximab treatment experienced significantly fewer relapses within 1 year compared to those treated with cyclophosphamide (pooled WMD=-0.70, 95%CrI: -1.10, -0.25) (**Figure 3A**). According to the league table, CsA (pooled WMD=0.94, 95%CrI: 0.50, 1.38), cyclophosphamide (pooled WMD=0.70, 95%CrI: 0.34, 1.05) and MMF (pooled WMD=0.55, 95%CrI: 0.18, 0.91) were associated with significantly more relapses within 1 year, as compared with rituximab (**Table 2**). The rank probabilities indicated that rituximab had the highest likelihood (92.44%) to be associated with the fewest relapses within 1 year (**Table 3**).

**Table 2:** League table of different immunosuppressive agents for various outcomes in pediatric FRSDNS.

Relapses within 1 year					
CsA	-0.24 (-0.61, 0.12)	-0.39 (-0.7, -0.08)	-0.94 (-1.38, -0.5)	-0.68 (-1, -0.35)	
0.24 (-0.12, 0.61)	Cyclophosphamide	-0.15 (-0.48, 0.19)	-0.7 (-1.05, -0.34)	-0.44 (-0.77, -0.1)	
0.39 (0.08, 0.7)	0.15 (-0.19, 0.48)	MMF	-0.55 (-0.91, -0.18)	-0.29 (-0.41, -0.16)	
0.94 (0.5, 1.38)	0.7 (0.34, 1.05)	0.55 (0.18, 0.91)	Rituximab	0.26 (-0.1, 0.61)	
0.68 (0.35, 1)	0.44 (0.1, 0.77)	0.29 (0.16, 0.41)	-0.26 (-0.61, 0.1)	Tacrolimus	
Mean cumulative exposure to corticosteroids					
CsA	-61.48 (-112.85, -9.95)	-49.88 (-89.07, -10.92)	-127.9 (-169.92, -85.91)	-75.94 (-115.97, -36.31)	
61.48 (9.95, 112.85)	Cyclophosphamide	11.42 (-22.29, 45.6)	-66.47 (-97.46, -35.32)	-14.64 (-47.33, 18.29)	
49.88 (10.92, 89.07)	-11.42 (-45.6, 22.29)	MMF	-77.97 (-93.89, -62.07)	-26.09 (-34.52, -17.67)	
127.9 (85.91, 169.92)	66.47 (35.32, 97.46)	77.97 (62.07, 93.89)	Rituximab	51.86 (38.48, 65.27)	
75.94 (36.31, 115.97)	14.64 (-18.29, 47.33)	26.09 (17.67, 34.52)	-51.86 (-65.27, -38.48)	Tacrolimus	
Patients with treatment failure at 1 year					
CsA	1.08 (0.03, 40.19)	2.21 (0.15, 64.24)	0.58 (0.02, 28.2)	2.16 (0.11, 74.23)	
0.93 (0.02, 35.38)	Cyclophosphamide	2.03 (0.14, 66.91)	0.54 (0.02, 29.05)	1.99 (0.1, 76.21)	
0.45 (0.02, 6.57)	0.49 (0.01, 7.27)	MMF	0.26 (0.02, 1.95)	0.98 (0.28, 3.13)	
1.72 (0.04, 59.31)	1.84 (0.03, 65.32)	3.78 (0.51, 40)	Rituximab	3.6 (0.74, 27.43)	
0.46 (0.01, 8.96)	0.5 (0.01, 9.84)	1.02 (0.32, 3.6)	0.28 (0.04, 1.35)	Tacrolimus	
Relapse-free survival during 1 year					
CsA	0.1 (0, 5.07)	0.18 (0.01, 1.83)	0.38 (0.01, 16.02)	0.4 (0.02, 6.15)	0.4 (0.04, 3.68)
10.22 (0.2, 579.48)	Cyclophosphamide	1.8 (0.04, 73.49)	3.86 (0.08, 185.7)	4.07 (0.24, 77.46)	4.02 (0.15, 110.06)
5.65 (0.55, 67.72)	0.56 (0.01, 23.35)	MMF	2.14 (0.07, 70.85)	2.28 (0.23, 24.5)	2.24 (0.41, 12.67)
2.64 (0.06, 116.19)	0.26 (0.01, 11.85)	0.47 (0.01, 14.14)	ofatumumab	1.06 (0.08, 13.57)	1.04 (0.05, 21.05)
2.48 (0.16, 40.84)	0.25 (0.01, 4.23)	0.44 (0.04, 4.35)	0.94 (0.07, 12.13)	Rituximab	0.98 (0.2, 4.68)
2.53 (0.27, 25.83)	0.25 (0.01, 6.65)	0.45 (0.08, 2.43)	0.96 (0.05, 19.84)	1.02 (0.21, 4.98)	Tacrolimus

FRSDNS, frequently relapsing or steroid-dependent nephrotic syndrome; CsA; cyclosporine A; MMF, mycophenolate mofetil.

**Table 3:** Rank probabilities of different immunosuppressive agents for various outcomes in pediatric FRSDNS.

Relapses within 1 year						
	[1]	[2]	[3]	[4]	[5]	
CsA	0.90274	0.09368	0.00348	0.000025	0.000075	
Cyclophosphamide	0.09318	0.71464	0.18669	0.00547	0.00002	
MMF	0.00408	0.191625	0.80243	0.001865	0	
Rituximab	0	0.00002	0.00188	0.07369	0.92441	
Tacrolimus	0	0.000035	0.00552	0.91895	0.075495	
Mean cumulative exposure to corticosteroids						
	[1]	[2]	[3]	[4]	[5]	
CsA	0.986515	0.011105	0.002275	0.0001	0.000005	
Cyclophosphamide	0.008905	0.24587	0.55309	0.19207	0.000065	
MMF	0.00458	0.743	0.25242	0	0	
Rituximab	0	0.000005	0	0.00007	0.999925	
Tacrolimus	0	0.00002	0.192215	0.80776	0.000005	
Patients with treatment failure at 1 year						
	[1]	[2]	[3]	[4]	[5]	
CsA	0.17561	0.127325	0.17456	0.2545	0.268005	
Cyclophosphamide	0.200145	0.12728	0.17816	0.241895	0.25252	
MMF	0.278395	0.3803	0.24477	0.083805	0.01273	
Rituximab	0.028845	0.05511	0.17117	0.28509	0.459785	
Tacrolimus	0.317005	0.309985	0.23134	0.13471	0.00696	
Relapse-free survival during 1 year						
	[1]	[2]	[3]	[4]	[5]	[6]
CsA	0.57932	0.169205	0.099395	0.08275	0.051865	0.017465
Cyclophosphamide	0.05076	0.062065	0.064125	0.09008	0.184065	0.548905
MMF	0.01574	0.074865	0.112445	0.16819	0.348745	0.280015
ofatumumab	0.214885	0.189505	0.127075	0.153365	0.193065	0.122105
Rituximab	0.0826	0.23966	0.304845	0.24669	0.111255	0.01495
Tacrolimus	0.056695	0.2647	0.292115	0.258925	0.111005	0.01656

FRSDNS, frequently relapsing or steroid-dependent nephrotic syndrome; CsA; cyclosporine A; MMF, mycophenolate mofetil.

#### Mean cumulative exposure to corticosteroids

Five studies (9, 10, 18, 22, 23) including 459 patients assessed 5 immunosuppressive agents (cyclophosphamide, rituximab, tacrolimus, MMF, and CsA) in terms of the mean cumulative exposure to corticosteroids. A larger number of studies focused on the direct comparison between rituximab and tacrolimus, with a greater number of children receiving treatment with tacrolimus (**Figure 2B**). The forest plot illustrated that the mean cumulative exposure to corticosteroids was significantly lower in children treated with rituximab compared to those in the cyclophosphamide group (pooled WMD=-66.00, 95%CrI: -100.00, -32.00); patients treated with tacrolimus experienced a significantly higher mean cumulative exposure to corticosteroids compared to those receiving rituximab (pooled WMD=52.00, 95%CrI: 38.00, 65.00) (**Figure 3B**). The league table showed that the CsA (pooled WMD=127.90, 95%CrI: 85.91, 169.92), cyclophosphamide (pooled WMD=66.47, 95%CrI: 35.32, 97.46), and MMF (pooled WMD=77.97, 95%CrI: 62.07, 93.89) groups had significantly greater mean cumulative exposure to corticosteroids than the rituximab group (**Table 2**). According to the rank probabilities, patients treated with rituximab were the most likely, with a 99.99% probability, to have the lowest mean cumulative exposure to corticosteroids (**Table 3**).

#### Patients with treatment failure at 1 year

Treatment failure at 1 year was estimated in 3 studies (21–23) with 371 patients, and 5 immunosuppressive agents were compared: cyclophosphamide, rituximab, tacrolimus, MMF, and CsA. MMF was a more commonly applied drug (**Figure 2C**). The league table indicated that there was no significant difference in the number of patients experiencing treatment failure at 1 year between the groups treated with cyclophosphamide, rituximab, tacrolimus, MMF, and CsA (**Table 2**). The rank probabilities suggested that rituximab had the greatest likelihood (45.98%) to be associated with the smallest number of patients with treatment failure at 1 year (**Table 3**).

#### Relapse-free survival during 1 year

Nine studies (11, 15, 18–20, 22, 23, 25, 26) with 796 patients evaluated relapse-free survival during 1 year after treatment with 6 immunosuppressive agents (cyclophosphamide, rituximab, tacrolimus, MMF, CsA, and ofatumumab). Tacrolimus was administered to a larger number of children, and a higher number of studies directly compared tacrolimus with rituximab (**Figure 2D**). The forest plot (**Figure 3C**) and league table (**Table 2**) exhibited no significant difference in relapse-free survival during 1 year among the 6 medications. The rank probabilities indicated that, among children, those treated with CsA had the highest likelihood (57.93%) of achieving the greatest relapse-free survival over 1 year, followed closely by rituximab (30.48%) and tacrolimus (26.47%) (**Table 3**).

#### Adverse events

In the study of Wang et al. (9), 108 adverse events were recorded within 1 year: 24 in the rituximab group, 34 in the tacrolimus group, and 50 in the cyclophosphamide group (*P* = 0.327). The difference was primarily caused by infection. The incidence of infection with cyclophosphamide was almost twice that of rituximab and tacrolimus (*P* = 0.001). Ofatumumab and rituximab had similar adverse effects according to Ravani et al. (11). Both agents did not bring side effects during infusion, with most limited to pruritus or erythema. Wang et al. (20) illustrated no significant difference in the incidence of adverse events, such as infection, gastrointestinal symptoms, acute kidney injury, and neutropenia between patients using tacrolimus and MMF. The most common adverse event was infection, which was found in four (11.8%) MMF patients and three (7.89%) tacrolimus patients.

## Discussion

This updated systematic review and network meta-analysis comprehensively assessed and compared the effectiveness of different immunosuppressive agents in pediatric patients with FRSDNS by incorporating studies with head-to-head comparisons of these agents. Rituximab emerged as a potentially superior medication in terms of reducing relapses within 1 year, minimizing mean cumulative corticosteroid exposure, and reducing treatment failure rates at 1 year. Furthermore, children with FRSDNS treated with rituximab may experience better relapse-free survival over the course of 1 year. These findings have implications for clinicians when selecting immunosuppressive agents for the treatment of pediatric FRSDNS and suggest that rituximab should be considered due to its favorable effectiveness.

A review by Larkins et al. (7) assessed non-corticosteroid immunosuppressive drugs in pediatric SSNS and reported that rituximab was an important adjunctive therapy for SDNS, and greatly reduces recurrences in children with relapsing SSNS. For FRSDNS in children, Tan et al. (6) performed a network meta-analysis for efficacy and acceptability of immunosuppressive medications in FRSDNS among children, and concluded that cyclophosphamide may be a preferable agent; chlorambucil and rituximab may also be suitable in treating FRSDNS. The above meta-analysis used placebo and no treatment as common comparators, and merely 8 out of 26 studies provided head-to-head comparisons of different immunosuppressive agents, which indicated insufficient evidence of direct comparison between immunosuppressive drugs. Besides, only the relapse rate was evaluated for efficacy. The present network meta-analysis utilized the latest direct comparison of various immunosuppressive agents to assess and rank the effectiveness of these agents and illustrated that considering relapses within 1 year, the mean cumulative exposure to corticosteroids, the number of patients with treatment failure at 1 year, and relapse-free survival during 1 year, rituximab may be the most favorable immunosuppressive drug for children with FRSDNS. Although CsA was most likely to be associated with the highest relapse-free survival during 1 year, no significant difference was found between rituximab and CsA. Rituximab is identified as a standard treatment for childhood-onset complicated FRSDNS (27, 28). A previous review showed the efficacy of rituximab in complicated FRSDNS (29). Rituximab, a monoclonal anti-CD20 antibody, has been shown in several studies to prolong clinical remission and reduce glucocorticoid dosages in children with FRSDNS (30, 31). Rituximab therapy causes B cell depletion by apoptosis, antibody-dependent cellular cytotoxicity or phagocytosis, reducing interactions between B cells and T cells, which perhaps prevents relapses in patients with FRSDNS. Besides, rituximab may improve the number and function of regulatory T (Treg) cells which have been demonstrated to cause remission in individuals with nephrotic syndrome, thus bringing better prognosis to patients (32, 33). Rituximab has been considered a potential substitute for cyclophosphamide and calcineurin inhibitors in SDNS (34). Recent evidence showed an advantageous impact of rituximab in the treatment of FRSDNS, reducing the need for steroids and other immunosuppressive agents to the lowest possible level (35, 36).

Therapeutic options for FRSDNS depend not only on efficacy, but also on safety factors. Rituximab is often well tolerated with very low occurrences of serious adverse events, making it an appealing treatment choice in patients with autoimmune or immune-mediated diseases (34). In the current analysis, with limited data from the included studies, the qualitative description was conducted, and rituximab was shown to have no relationship with serious side effects, and seemed to have comparable adverse influences to ofatumumab and tacrolimus, which indicated that rituximab may be a safe and feasible agent for children with FRSDNS. These results necessitate future studies for validation. Additionally, for many families, the ability to achieve disease remission with a single course of intravenous treatment without the requirement for maintenance oral medication intake is the main practical reason in favor of rituximab. Of note, the total cost of therapy is a concern, especially in developing countries. In a 12-month research, the cost of two doses of rituximab was up to 20% lower than the total cost for even the generic tacrolimus formulations adopted in India (23). A study for FRSDNS in Japan confirmed the cost-effectiveness of rituximab, with the total medical expenses declining from 2923 USD to 1280 USD per month after rituximab use (37). Iorember et al. (38) reported that the annualized healthcare expenditure for the rituximab group was 197031 USD compared with 189857 USD for the CNI group (*P* > 0.05). This evidence may further support the clinical application of rituximab.

With the updated head-to-head comparison of different immunosuppressive agents, rituximab may be a beneficial option in treating FRSDNS. Apart from efficacy and safety, nephrologists could take this agent into account based on their clinical experience, patient characteristics, and costs in clinical practice. Some limitations should be acknowledged in result interpretation. First, most included studies were cohort studies, which may negatively affect the quality of evidence in this analysis. Second, some outcomes, such as the number of patients with treatment failure at 1 year, incorporated a relatively small number of studies and patients, probably lowering the accuracy of the results. Dosages and administration routes of immunosuppressive agents may play a role in their efficacy, which was not considered in the present work. More studies are warranted to explore this point.

## Conclusion

Rituximab may be the most effective agent regarding relapses within 1 year, the mean cumulative exposure to corticosteroids, and treatment failure at 1 year; relatively better relapse-free survival during 1 year may be obtained after rituximab use in FRSDNS children. It is necessary to have future investigations to clinically verify the role of rituximab.

## Data availability statement

The raw data supporting the conclusions of this article will be made available by the authors, without undue reservation.

## Author contributions

YuZ: Conceptualization, Funding acquisition, Supervision, Writing – original draft, Writing – review & editing. JC: Data curation, Formal analysis, Investigation, Methodology, Writing – review & editing. YaZ: Data curation, Formal analysis, Investigation, Methodology, Writing – review & editing. XW: Data curation, Formal analysis, Investigation, Methodology, Writing – review & editing. JW: Conceptualization, Supervision, Writing – original draft, Writing – review & editing.
